# Variations in hip fracture inpatient care in Japan, Korea, and Taiwan: an analysis of health administrative data

**DOI:** 10.1186/s12913-021-06621-y

**Published:** 2021-07-13

**Authors:** Hongsoo Kim, Shou-Hsia Cheng, Hayato Yamana, Seyune Lee, Nan-He Yoon, Yi-Chieh Lin, Kiyohide Fushimi, Hideo Yasunaga

**Affiliations:** 1 Graduate School of Public Health Department of Public Health Science, Institute of Health and Environment, & Institute of Aging, Seoul National University, Seoul, 08826 South Korea; 2 Institute of Health Policy and Management, College of Public Health, Population Health Research Center, National Taiwan University, Taipei, Taiwan; 3 Department of Health Services Research, Graduate School of Medicine, The University of Tokyo, Tokyo, Japan; 4 Graduate School of Public Health, Department of Public Health Sciences, Seoul National University, Seoul, South Korea; 5 Division of Social Welfare and Health Administration, Wonkwang University, Iksan, Jeonbuk South Korea; 6 Institute of Health Policy and Management, College of Public Health, National Taiwan University, Taipei, Taiwan; 7 Department of Health Policy and Informatics, Tokyo Medical and Dental University Graduate School, Tokyo, Japan; 8 Department of Clinical Epidemiology and Health Economics, School of Public Health, The University of Tokyo, Tokyo, Japan

## Abstract

**Background:**

Little is known about hip fracture inpatient care in East Asia. This study examined the characteristics of patients, hospitals, and regions associated with delivery of hip fracture surgeries across Japan, Korea, and Taiwan. We also analyzed and compared how the resource use and a short-term outcome of the care in index hospitals varied according to factors in the respective health systems.

**Methods:**

We developed comparable, nationwide, individual-level health insurance claims datasets linked with hospital- and regional-level statistics across the health systems using common protocols. Generalized linear multi-level analyses were conducted on length of stay (LOS) and total cost of index hospitalization as well as inpatient death.

**Results:**

The majority of patients were female and aged 75 or older. The standardized LOS of the hospitalization for hip fracture surgery was 32.5 (S.D. = 18.7) days in Japan, 24.7 (S.D. = 12.4) days in Korea, and 7.1 (S.D. = 2.9) days in Taiwan. The total cost per admission also widely varied across the systems. Hospitals with a high volume of hip fracture surgeries had a lower LOS across all three systems, while other factors associated with LOS and total cost varied across countries.

**Conclusion:**

There were wide variations in resource use for hip fracture surgery in the index hospital within and across the three health systems with similar social health insurance schemes in East Asia. Further investigations into the large variations are necessary, along with efforts to overcome the methodological challenges of international comparisons of health system performance.

## Background

In the face of ever-increasing complex care needs and expenditures during an economic downturn, sustainable health systems with a high quality of care are a top priority for countries with aging populations. Japan, South Korea (hereafter Korea), and Taiwan are high-income economies in East Asia with social health insurance (SHI)-based health systems known for universal coverage with relatively good performance, such as long life expectancy and easy access to care with a low copayment [1–4]. Japan and Korea have the largest number of acute care hospital beds and the longest lengths of stay (LOS) in the OECD, and Taiwan also has a higher number of beds than the OECD average [4]. Facing rapidly aging populations with higher disease burdens, the sustainability of these countries’ current inpatient-centered health care delivery is being challenged, and reforms to improve system efficiency have been implemented, including payment-system reforms [3, 5–8]. Inpatient care is also often the target of major health care reforms in most developed countries, as it is the most intense and expensive care [9–13]. International comparisons of health systems focusing on inpatient care have been widely conducted led by the OECD and WHO European Health Observatory [4, 14], but little work has been done in Asia.

In the OECD Health Care Quality Indicators (HCQI) project, an international quality initiative among member countries, the waiting time for hip fracture care is a hospital performance indicator [15]. Waiting time, however, is rarely an issue for the three East Asian SHI systems examined here, and the indicator has not been reported by Japan or Korea. EuroHOPE is another multinational project among six European countries [16], and hip fracture surgery is one of the clinical conditions the project has selected for health system performance comparisons of inpatient care using patient-level registry data. A study by the EuroHOPE team reported the standardized average length of stay (ALOS) of hip fracture surgery patients at the first (index) hospital varied from 9.8 days in Norway to 18.9 days in Italy [17]. Another report also found systematic country-level variations in financial performance: the costs for hip fracture surgery were 29–37% lower in Finland and Norway, but 14% higher in Italy, compared to Sweden [16].

The purpose of this study is to investigate variations in health system performance in three SHI systems in East Asia using comparable micro health insurance data. We selected hip fracture as the tracer condition for our system performance comparison for several reasons. Hip fracture is common in aging populations, and its incidence is expected to increase [18–20]; the number of hip fractures will increase by more than 2.28 times by 2050, and the direct cost for hip fractures will increase by 1.59-fold due to population change in Asia [20, 21]. Hip fractures also have a significant negative impact on mobility and quality of life, which can increase the risk of disability and death [20, 22–24]. In addition, the high prevalence of hip fracture surgeries for the oldest old as well as the younger old has raised attention to quality of care and the financial burden to health systems [25, 26].

With similar rationales, existing studies have widely examined the quality of care for hip fracture patients using clinical and financial measures including LOS, cost, complications, and mortality among middle-aged and/or older populations. They have reported on variations in patient outcomes and their attributes at the patient and also hospital levels [27–29]. Organizational- and national-level efforts have been made in order to improve clinical quality and decrease unwarranted variations through practice guidelines and quality-improvement initiatives [27, 30]. Yet almost all are single country-based studies, and only a few studies have examined variations in care for hip fracture patients at the health system level across countries, most of which were somewhat descriptive [21, 30, 31].

This study aimed to examine the variations in LOS and related costs for inpatient hip fracture surgery care in Japan, Korea, and Taiwan. We did not intend to assess the performance of the whole health system using this one condition. Rather, we examined the variations in resource use (efficiency) in inpatient care provision in the most similar SHI systems in East Asia.

## Methods

### Study population and database development

#### The sample

The study sample consists of people aged 50 or older undergoing hip fracture surgery based on the OECD HCQI criteria [15]. We used data from 2016, which was the most recent available data across the three health systems. We first identified patients who were hospitalized in 2016 with hip fracture as the principal diagnosis (WHO International Classification of Diseases, 10th edition code S72.0-2) and then further selected those with three common hip fracture surgery procedures: internal fixation, hemiarthroplasty, and total hip arthroplasty. The procedures for the study were determined based on existing studies [27, 28, 32, 33] and consultation with clinical experts. We excluded patients with a hip fracture admission during the 365 days immediately prior to the index admission, those under 50 years at the time of the index admission, and those with no hip fracture surgery or with another type of procedure that this study did not focus on. We only included the first surgery admission if a patient had multiple hip fracture surgeries in the year observed, although such a case was uncommon. The sample selection was based on a common protocol that had been developed and shared among the research teams across the three health systems.

#### Databases

*Japan:* We used the DPC database to extract patient-level data. The Diagnosis Procedure Combination (DPC) database is a nationwide inpatient database of approximately 1000 participating DPC hospitals and covers approximately 50% of all acute-care admission in Japan. The database includes discharge abstract and administrative claims data including diagnoses, comorbidities, surgical procedures, and drugs. Details of the database are provided elsewhere [5]. Additionally, we used hospital characteristics from Reporting System for Functions of Medical Institutions and Survey of Medical Institutions [34]. Regional characteristics were obtained Vital Statistics and other publicly available data sources.

*Republic of Korea:* We developed an analytic database based on data provided by the National Health Insurance Data Sharing Service run by the National Health Insurance Services (NHIS), the single public health insurer with universal coverage in Korea. The NHIS collects and maintains nearly all of the health and health care utilization data of all Koreans across their lifetime and across various care settings. In order to promote evidence-based health services and policy decision-making, the NHIS recently began to provide de-identified health administrative data to researchers for public use. The research database consists of micro-level health administration data and detailed claims data received from providers; it contains patient clinical and utilization data combined with the socio-demographic data of beneficiaries using a scrambled unique identifier [35]. We also collected regional data from the Korean National Health Insurance statistical yearbook (number of physicians and hospital beds), the Long-Term Care Insurance statistical yearbook (number of home care facilities), census data (% of elderly living alone), regional administrative statistics (social welfare expenditures), and OECD regional statistics (disposable income).

*Taiwan:* Data for this study came from the Health and Welfare Data Science Center of the Ministry of Health and Welfare in Taiwan. We submitted application to the center to obtain individual level records which were retrieved from multiple national databases and linked by a scrambled identification number for every resident in Taiwan. The essential dataset we have applied for this study was the National Health Insurance Research Database which included health care utilization variables (e.g., physician visits, hospitalization, diagnoses, procedures, etc.), expense for every service item, enrollees’ demographic variables, and contracted providers’ characteristics [36]. Under the compulsory universal coverage, more than 99% of the residents in Taiwan are enrolled and the dataset we obtained is comprehensive and representative. We also retrieved the mortality records from the Death Registry file as well as region-level data and linked all the records in our study dataset. Statistical analysis was conducted inside the data science center under close monitoring for information safety and privacy protection.

### Analytic framework & variables

The key outcome variables of this study were LOS and total cost. *LOS* was the total number of consecutive days of stay in the index (first) acute-care hospital where a hip fracture surgery was conducted. LOS counted the entire period from admission day to discharge day. *Total cost* in Korea and Taiwan refers to the sum of expenditures during the entire period of an index hospitalization for the surgery; the total cost included the expense reimbursed by the national health insurance services and the copayment paid by the patient (beneficiary). For Japan, the cost was calculated as the sum of fee-for-service (FFS) equivalent costs of all services provided during a hospitalization.

Based on the literature, patient, hospital, and regional factors associated with the outcome variables were included in the analytic models. Socio-demographic characteristics of patients included age, sex, and household income. Clinical complexity was measured by the Charlson Comorbidity Index (CCI) [37], and surgical procedure type was also included. Among hospital characteristics, hospital size was measured by the number of hospital beds and service volume by the number of hip fracture surgery patients in a hospital, counted and divided into quartiles. Others included location (urban/rural), ownership (private/public), teaching status (yes/no). In Taiwan, since information on bed numbers was not available, hospital accreditation level was used as a proxy.

Regional characteristics of the areas where hospitals were located were also included in the analytic model. Disposable regional income per person as a proxy of regional economic status was collected and transformed into USD, 2010 PPP. Regional competition, or concentration, was calculated using the Herfindahl-Hirschman Index (HHI) [38]. We also collected data on the number of physicians and hospital beds per 1000 persons and the number of home care facilities per 10,000 persons to examine health and welfare service capacity (resources) at the regional level. Lastly, data on the proportion of older people living in the community independently and the proportion of social welfare expenditure to total expenditure at the regional level were included in the analysis.

### Analysis

Descriptive statistics were calculated to summarize the general patient and hospital (and regional) characteristics in each system. The outcome variables including LOS and cost were age- and sex-standardized [39, 40] with the Korean population as the reference group. Multivariate, multi-level analyses were conducted to examine the factors associated with LOS and total cost. We analyzed the factors associated with the outcome variables while adjusting for hospital-level characteristics as well as patient-level characteristics nested in each index hospital where patients received a hip fracture surgery. We computed intraclass correlation coefficients (ICCs) to examine the proportion of variance in each outcome variable that was attributable to the hospital level [41–44]. We could not conduct multivariate analyses for an inpatient mortality variable as inpatient death during a hospitalization for a hip fracture surgery was a rare event (occurred in about 1% or less of the patients in the sample) across all three systems.

As access to the data was only allowed for the researchers in each respective country, each of the three teams developed and analyzed its own data using the same protocol and common analytic codes, which were initially developed by the PI’s team in Korea and finalized with the research teams in Japan and Taiwan through multiple rounds of reviews and discussions. The data work was done locally, and only the final tables were shared. Minor adjustments to the codes due to data availability (e.g., no income data in Japan) were allowed after discussion by the whole team.

## Results

Table 1 presents the demographic and clinical profiles of patients and the outcome variables of the study. A total of 61,662, 23,226, and 15,235 patients had at least one hip fracture surgery in Japan, Korea, and Taiwan, respectively, in 2016. The majority of the patients were women, and the mean age of the patients was 82.8 in Japan, 78.7 in Korea, and 78.3 in Taiwan. Hypertension, diabetes, and dementia were the most prevalent chronic conditions in all three health systems. Among the types of surgery, internal fixation was the most common in Japan and Korea, and hemi-arthroplasty was the most common in Taiwan.

**Table 1:** Characteristics of hip fracture surgery patients in Japan, Korea, and Taiwan

		Japan		Korea		Taiwan	
		*n* (mean)	% (SD)	*n* (mean)	% (SD)	*n* (mean)	% (SD)
Total		61,662		23,226		15,235	
Sex	Male	13,172	21.36	6254	26.93	5418	35.56
	Female	48,490	78.64	16,972	73.07	9817	64.44
Age	mean (SD)	82.82	9.24	78.71	9.41	78.25	10.50
	50–64	2971	4.82	2197	9.46	1915	12.57
	65–74	7548	12.24	3949	17.00	2822	18.52
	75–84	20,587	33.39	10,577	45.54	5663	37.17
	85 +	30,556	49.55	6503	28.00	4835	31.74
Household income level	Q1			5627	24.63	189	1.24
	Q2			5827	25.51	5472	35.92
	Q3			5195	22.74	5779	37.93
	Q4			6195	27.12	3795	24.91
Comorbidity							
Charlson index	mean (SD)	1.00	1.31	0.75	0.99	0.94	1.55
	0	36,332	58.92	11,634	50.09	8526	55.96
	1	4596	7.45	7740	33.32	3136	20.58
	2	15,632	25.35	2664	11.47	1861	12.22
	3	2054	3.33	806	3.47	868	5.70
	4+	3048	4.94	382	1.64	844	5.54
Comorbid diseases	Hypertension	23,497	38.11	7418	31.94	7554	49.58
	Coronary artery disease	5260	8.53	1215	5.23	748	4.91
	Diabetes mellitus	11,561	18.75	5007	21.56	4099	26.91
	Dementia	11,499	18.65	1847	7.95	872	5.72
	Stroke	6787	11.01	1651	7.11	818	5.37
Surgical intervention/ Procedure	Internal fixation	40,406	65.53	12,322	53.05	7476	49.07
	Hemiarthroplasty	20,587	33.39	9911	42.67	7706	50.58
	Total hip arthroplasty	669	1.08	993	4.28	53	0.35
Age- and sex-adjusted length of stay	mean (SD)	32.5	18.7	24.7	12.4	7.1	2.9
Age- and sex-adjusted total cost per person^a^	mean (SD)	14,808.0	5365.6	7836.7	2924.3	3771.2	1239.5
Age- and sex-adjusted average cost per person per day^a^	mean (SD)	522.8	175.1	362.6	161.1	581.3	230.0
Inpatient mortality	*n* (%)	644	1.04	190	0.82	88	0.58

^a^USD PPP, 2010

As for the outcomes of interest, the age- and sex-standardized average LOS of hip fracture surgery patients in the index hospitals where patients underwent the surgery was 32.5 days in Japan, 24.7 days in Korea, and 7.1 days in Taiwan. The age- and sex-standardized average total cost for hip fracture surgery per person was highest in Japan (14,808.0 USD), followed by Korea (7836.7 USD) and Taiwan (3771.2 USD). The standardized average cost per day per person was highest in Taiwan (581.3 USD), followed by Japan (522.8 USD) and Korea (362.6 USD). In-hospital mortality was quite low in all three health systems—Japan (1.04%) followed by Korea (0.82%) and Taiwan (0.58%).

Table 2 includes hospital and regional characteristics. The majority of the index hospitals were located in urban areas in Korea and Taiwan, while more than half were located in rural areas in Japan. Most of the hospitals were private and teaching hospitals in all three systems. Regional disposable income was the highest in Japan, followed by Korea and Taiwan. Market competition measured by the HHI was highest (lowest HHI score) in Korea, followed by Japan and Taiwan. The proportion of older people living alone in the community was highest in Japan at 17.75% followed by Korea (7.5%), and lowest in Taiwan (2.7%). The number of physicians in the region was relatively similar in all three systems (2.4 – 2.6 physicians per 1000 persons). The number of hospital beds and home care facilities varied across the countries.

**Table 2:** Characteristics of hospitals where hip fracture surgery patients were admitted in Japan, Korea, and Taiwan

		Japan		Korea		Taiwan	
		*n* (mean)	% (SD)	*n* (mean)	% (SD)	*n* (mean)	% (SD)
Total		906		881		226	
Location	Urban	440	48.57	569	64.59	170	75.22
	Rural	466	51.43	312	35.41	56	24.78
Ownership	Public	343	37.86	37	4.2	60	26.55
	Private/Other	563	62.14	844	95.80	166	73.45
Teaching hospital	Yes	77	8.50	63	7.15	104	46.02
	No	829	91.50	818	92.85	122	53.98
No. of hosp. Beds	30–99	26	2.87	272	30.87	10	4.42
	100–399	510	56.29	505	57.32	39	17.26
	400+	370	40.84	104	11.8	177	78.32
No. of hip fracture patients	Q1	232	25.61	187	21.23	44	19.47
	Q2	223	24.61	236	26.79	60	26.55
	Q3	225	24.83	230	26.11	60	26.55
	Q4	226	24.94	228	25.88	62	27.43
Regional characteristics	Disposable income (USD, 2010 PPP)	19,971	2205	16,905	1507	15,039	1633
(for hospital location)	Herfindahl-Hirschman Index	766.62	631.12	661.38	505.44	1401.84	972.60
	No. of hospital with low HHI (<median)	696	76.82	673	76.39	100	44.25
	No. of hospitals with high HHI (≥median)	210	23.18	208	23.61	126	55.75
	% elderly living alone	17.75	3.65	7.46	2.49	2.67	2.67
	No. of physicians per 1000 persons	2.46	0.42	2.56	0.57	2.35	0.61
	No. of hospital beds per 1000 persons	13.08	3.44	15.21	5.19	7.21	1.91
	No. of home care facilities per 10,000 persons	16.08	3.85	2.97	0.74	0.37	0.28
	% social welfare expenditure	16.76	4.12	31.14	8.25	13.71	1.62

Table 3 presents factors associated with the LOS of hip fracture surgery patients in the index hospitals. LOS was longer among people who were older and had comorbidities reflecting clinical complexities measured by the CCI. While adjusting for patient characteristics, a higher volume of surgery was associated with a shorter LOS across all three health systems. As for hospital bed size, compared to small-size hospitals, the LOS of hip fracture patients was significantly longer in middle-size hospitals in Korea and in both middle- and large-size hospitals in Taiwan. Unlike in Korea and Taiwan, the LOS for hospitals with 400 or more beds in Japan was about 8.6 days shorter than hospitals with 100 or fewer beds. In Japan, LOS was also significantly shorter in urban, public, and/or teaching hospitals than in their counterparts in that country. The proportion of variance in LOS that was attributable to the hospital level, as described by the ICC, was highest in Japan (30.6%), followed by Korea (23.3%) and Taiwan (14.2%).

**Table 3:** Factors associated with length of stay for hip fracture surgery patients in Japan, Korea, and Taiwan^a^

		Japan			Korea			Taiwan		
		B	SE	*p*	B	SE	*p*	B	SE	*p*
*Individual characteristics*										
Sex	Male	(ref.)	(ref.)	(ref.)	(ref.)	(ref.)	(ref.)	(ref.)	(ref.)	(ref.)
	Female	−0.910	0.154	<0.001	1.249	0.159	<0.001	−0.126	0.048	0.008
Age	50–64	(ref.)	(ref.)	(ref.)	(ref.)	(ref.)	(ref.)	(ref.)	(ref.)	(ref.)
	65–74	1.454	0.336	<0.001	0.128	0.280	0.648	0.311	0.083	<0.001
	75–84	2.301	0.307	<0.001	0.197	0.254	0.438	0.696	0.075	<0.001
	85 +	1.232	0.304	<0.001	−0.313	0.269	0.246	1.045	0.077	<0.001
Household income level	Q1				(ref.)	(ref.)	(ref.)	(ref.)	(ref.)	(ref.)
	Q2				−0.341	0.192	0.076	−0.211	0.206	0.304
	Q3				−0.131	0.198	0.510	−0.362	0.206	0.079
	Q4				0.273	0.192	0.155	−0.382	0.207	0.066
Charlson Comorbidity Index	0	(ref.)	(ref.)	(ref.)	(ref.)	(ref.)	(ref.)	(ref.)	(ref.)	(ref.)
	1	2.377	0.244	<0.001	0.323	0.157	0.039	0.500	0.058	<0.001
	2	−0.836	0.153	<0.001	1.380	0.229	<0.001	0.649	0.071	<0.001
	3	1.683	0.354	<0.001	3.176	0.383	<0.001	0.690	0.099	<0.001
	4+	−0.231	0.299	0.440	2.571	0.545	<0.001	0.825	0.100	<0.001
Surgical intervention/ Procedure	Internal fixation	(ref.)	(ref.)	(ref.)	(ref.)	(ref.)	(ref.)	(ref.)	(ref.)	(ref.)
	Hemiarthroplasty	0.251	0.135	0.063	−0.293	0.148	0.048	0.463	0.047	<0.001
	Total hip arthroplasty	2.351	0.625	<0.001	−0.415	0.375	0.269	1.070	0.386	0.006
*Hospital characteristics*										
Location	Urban	−2.789	0.987	0.005	−3.408	1.736	0.050	−0.422	0.409	0.304
	Rural	(ref.)	(ref.)	(ref.)	(ref.)	(ref.)	(ref.)	(ref.)	(ref.)	(ref.)
Ownership	Public	−3.265	0.771	<0.001	5.336	1.057	<0.001	−0.135	0.197	0.493
	Private/Other	(ref.)	(ref.)	(ref.)	(ref.)	(ref.)	(ref.)	(ref.)	(ref.)	(ref.)
Teaching hospital	Yes	−6.319	1.512	<0.001	−3.043	0.948	0.001	0.128	0.280	0.647
	No	(ref.)	(ref.)	(ref.)	(ref.)	(ref.)	(ref.)	(ref.)	(ref.)	(ref.)
No. of hosp. Beds	30–99	(ref.)	(ref.)	(ref.)	(ref.)	(ref.)	(ref.)	(ref.)	(ref.)	(ref.)
	100–399	−1.462	2.239	0.514	4.376	0.677	<0.001	1.976	0.620	0.002
	400+	−8.584	2.316	<0.001	1.745	1.049	0.097	2.311	0.611	<0.001
No. of hip fracture patients	Q1	(ref.)	(ref.)	(ref.)	(ref.)	(ref.)	(ref.)	(ref.)	(ref.)	(ref.)
	Q2	−2.258	1.064	0.034	−1.684	0.852	0.048	−0.919	0.361	0.011
	Q3	−4.930	1.077	<0.001	−0.536	0.895	0.550	−1.288	0.396	0.001
	Q4	−12.407	1.087	<0.001	−0.949	0.969	0.328	−1.354	0.413	0.001
Regional characteristics (for hospital location)	Disposable income (USD PPP, 2010)	0.255	0.271	0.347	−0.389	0.369	0.292	0.081	0.078	0.300
	Herfindahl-Hirschman index	2.385	1.075	0.027	1.489	0.636	0.019	−1.092	0.394	0.006
	% elderly living alone	−0.216	0.157	0.168	0.748	0.292	0.011	0.007	0.083	0.929
	No. of physicians per 1000 persons	2.576	1.374	0.061	−0.094	0.894	0.916	−0.922	0.543	0.091
	No. of hospital beds per 1000 persons	−0.124	0.197	0.528	−0.069	0.086	0.421	0.098	0.149	0.511
	No. of home care facilities per 10,000 persons	−0.145	0.111	0.192	−1.716	0.760	0.024	−0.402	0.867	0.643
	% social welfare expenditure	0.289	0.101	0.004	0.232	0.126	0.067	−0.010	0.096	0.918

^a^The intraclass correlation coefficient in each analytic model is as follows: Japan (0.306), Korea (0.233), and Taiwan (0.142)

Both old age and comorbidity increased the total cost (per admission) of hip fracture surgeries in the index hospitals in all three systems (Table 4). Patients with hemiarthroplasty or total hip arthroplasty spent more than those with internal fixation in all three systems. In all three health systems, there was no difference between urban and rural hospitals in terms of the total cost of inpatient care for hip fracture surgery patients during their index hospitalization. The total cost was lower in hospitals with the highest patient volume (the fourth quartile) in Japan compared to hospitals with the lowest volume. The total cost was lower in public than private hospitals in Japan and Taiwan; but in Korea the cost was higher in public hospitals, where LOS was also significantly longer than in private hospitals. The ICC was highest in Korea (29.8%), followed by Japan (13.8%) and Taiwan (9.0%).

**Table 4:** Factors associated with total cost of hip fracture surgery for patients during an index hospitalization in Japan, Korea, & Taiwan^a^

		Japan			Korea			Taiwan		
		B	SE	*p*	B	SE	*p*	B	SE	*P*
*Individual characteristics*										
Sex	Male	(ref.)	(ref.)	(ref.)	(ref.)	(ref.)	(ref.)	(ref.)	(ref.)	(ref.)
	Female	−0.026	0.003	<0.001	0.009	0.004	0.014	−0.009	0.004	0.021
Age	50–64	(ref.)	(ref.)	(ref.)	(ref.)	(ref.)	(ref.)	(ref.)	(ref.)	(ref.)
	65–74	0.019	0.007	0.005	0.075	0.007	<0.001	0.043	0.006	<0.001
	75–84	0.030	0.006	<0.001	0.117	0.006	<0.001	0.065	0.006	<0.001
	85 +	0.020	0.006	0.001	0.133	0.006	<0.001	0.096	0.006	<0.001
Household income level	Q1				(ref.)	(ref.)	(ref.)	(ref.)	(ref.)	(ref.)
	Q2				0.011	0.005	0.013	−0.014	0.016	0.382
	Q3				0.010	0.005	0.044	−0.016	0.016	0.303
	Q4				0.013	0.005	0.005	−0.027	0.016	0.098
Charlson Comorbidity Index	0	(ref.)	(ref.)	(ref.)	(ref.)	(ref.)	(ref.)	(ref.)	(ref.)	(ref.)
	1	0.072	0.005	<0.001	0.023	0.004	<0.001	0.045	0.005	<0.001
	2	0.007	0.003	0.039	0.075	0.005	<0.001	0.083	0.006	<0.001
	3	0.080	0.007	<0.001	0.155	0.009	<0.001	0.104	0.008	<0.001
	4+	0.027	0.006	<0.001	0.199	0.013	<0.001	0.105	0.008	<0.001
Surgical intervention/ Procedure	Internal fixation	(ref.)	(ref.)	(ref.)	(ref.)	(ref.)	(ref.)	(ref.)	(ref.)	(ref.)
	Hemiarthroplasty	0.248	0.003	<0.001	0.283	0.004	<0.001	0.461	0.004	<0.001
	Total hip arthroplasty	0.447	0.013	<0.001	0.412	0.009	<0.001	0.522	0.030	<0.001
*Hospital characteristics*										
Location	Urban	−0.021	0.013	0.104	0.006	0.048	0.907	−0.002	0.026	0.933
	Rural	(ref.)	(ref.)	(ref.)	(ref.)	(ref.)	(ref.)	(ref.)	(ref.)	(ref.)
Ownership	Public	−0.046	0.010	<0.001	0.256	0.029	<0.001	−0.034	0.012	0.006
	Private, others	(ref.)	(ref.)	(ref.)	(ref.)	(ref.)	(ref.)	(ref.)	(ref.)	(ref.)
Teaching hospital	Yes	0.036	0.020	0.074	0.034	0.027	0.206	−0.036	0.018	0.041
	No	(ref.)	(ref.)	(ref.)	(ref.)	(ref.)	(ref.)	(ref.)	(ref.)	(ref.)
No. of Hosp. beds	30–99	(ref.)	(ref.)	(ref.)	(ref.)	(ref.)	(ref.)	(ref.)	(ref.)	(ref.)
	100–399	0.000	0.030	0.996	0.151	0.018	<0.001	0.111	0.042	0.009
	400+	−0.057	0.031	0.064	0.225	0.029	<0.001	0.160	0.042	<0.001
No. of hip fracture patients	Q1	(ref.)	(ref.)	(ref.)	(ref.)	(ref.)	(ref.)	(ref.)	(ref.)	(ref.)
	Q2	−0.003	0.014	0.832	−0.014	0.022	0.521	−0.007	0.025	0.769
	Q3	−0.020	0.014	0.164	0.065	0.024	0.006	−0.037	0.027	0.175
	Q4	−0.105	0.014	<0.001	0.114	0.026	<0.001	0.010	0.028	0.719
Regional characteristics (where the hospital located)	Disposable income (USD PPP, 2010)	−0.001	0.004	0.724	0.005	0.010	0.625	−0.002	0.005	0.721
	Herfindahl-Hirschman index	0.027	0.014	0.052	0.045	0.018	0.011	−0.034	0.025	0.171
	% elderly lives alone	0.001	0.002	0.479	0.010	0.008	0.223	−0.003	0.005	0.605
	No. of physicians per 1000 persons	0.019	0.018	0.296	−0.006	0.025	0.816	−0.048	0.034	0.159
	No. of hospital beds per 1000 persons	−0.005	0.003	0.048	−0.001	0.002	0.647	0.007	0.009	0.450
	No. of home care facilities per 10,000 persons	−0.001	0.001	0.622	0.003	0.021	0.904	−0.009	0.055	0.872
	% social welfare expenditure	0.003	0.001	0.025	0.002	0.003	0.663	−0.001	0.006	0.815

^a^The intraclass correlation coefficient in each analytic model is as follows: Japan (0.138), Korea (0.298), and Taiwan (0.090)

## Discussion

The main purpose of this study was to assess the performance efficiency of three similar health systems in East Asia, i.e., Japan, Korea, and Taiwan. Using hip fracture surgery as a tracer condition, we found wide variations in the health care resources used for treating these patients. The ALOS ranged from 7.1 days to 32.5 days while the total cost admission ranged from 3771.2 USD to 14,808.0 USD. A few hospital-level or region-level common factors may account for the variations in this study. (For example, hospitals with a high volume of hip fracture surgeries had a lower LOS across all three systems).

In the typology of health system classification, Japan, Korea, and Taiwan are often categorized as SHI models with state regulation, societal financing, and private provision [1–3, 45]. We confirmed empirically that the profiles of patients receiving hip fracture surgery, a tracer condition of the study, were similar across the systems: a typical patient was female, aged around eighty, with one or more chronic conditions. This is also consistent with the profiles of hip fracture surgery patients in studies from the United States and other western countries [11, 27, 31, 46, 47]. As population aging and advances in healthcare technology continue, the number of the oldest old receiving complex surgeries will increase, and caring for these potentially high-need, high-cost patients will be a challenge across health systems [25].

While their patient profiles were similar, the age- and sex-adjusted LOSs widely varied. This suggests system factors, the approaches to treating potentially high-risk patients, widely differed between the three systems. Japan kept the surgical patients for more than one month in the index hospital where the patients had received hip fracture surgery; Korea, a little more than Three weeks; and Taiwan discharged patients within a week or so, which is similar to LOS in the United States [31, 48], although patient profiles were not exactly the same across existing studies.

The very long ALOS of hip fracture surgery patients in Japan and Korea is likely related to the countries’ high supply and capacity of acute care beds; the two countries had the highest numbers of hospital beds (13.050 and 12.270 per 1000 inhabitants) and longest ALOSs (16.2 and 7.5 days in Japan and Korea, respectively) among OECD countries in 2017 [4]. Different payment schemes and care delivery systems can also explain the different provider (hospital) behavior in the systems. Japan’s DPC is often compared with the diagnosis-related group (DRG) system, but the primary purpose of DPC is not cost-containment but rather standardization and quality improvement [49]. South Korea, meanwhile, still pays for hip fracture surgery with FFS schemes, which give hospitals incentive to keep patients longer and provide more services. The study results confirm the DRG payment scheme in Taiwan is a strong incentive for hospitals to discharge patients early, as has been reported by previous studies [50].

As for care delivery, Japanese hospitals tend to provide post-acute convalescence and rehabilitation care after surgery; such care would be necessary and could be more efficient in the end than immediate discharge to the community after surgery for people in their eighties and nineties at high risk for complications [51]. In Korea, introducing reforms to cut the number of hospital beds and ALOS has been difficult, as resistance comes from not only providers but also citizens, who are used to and pleased with easy access to inpatient care. The lack of a community-based post-acute care delivery system has also been a barrier for reforms, in response to which a major nationwide initiative to develop a community-based integrated care system was launched in 2018 [52, 53]. Similar to the situation in Korea, patients in Taiwan relied heavily on family member to receive rehabilitation services from the same of other hospitals after they were discharged [50].

Several hospital- and region-level factors that were associated with ALOS and the total cost of hip fracture surgery in the three health systems deserve discussion. First, hospitals with more experience in and higher demand for hip surgery procedures discharged patients more quickly; this volume-outcome effect was consistent across the three East Asian systems and also is consistent with the findings from existing literature [27, 28, 46]. Another interesting finding across all three systems was little variation between urban and rural areas in terms of the total cost of hip fracture surgery in the index hospitals; this demonstrates universal health coverage and the nationwide standardized payment schedules under their SHIs have effects on reducing health inequality.

In Japan, large public teaching hospitals located in urban areas discharged patients earlier than their counterparts. This may indicate the quality of care in these hospitals with more resources. Also, this may suggest that the postoperative transitional care is implemented more in these hospitals. In Korea, teaching hospitals with a high demand for the service, similar to Japan, discharged patients earlier (Table 3), as those hospitals had incentive to let patients out quickly right after the intensive service period. The long LOS and high cost of surgical patients in public hospitals in Korea were consistent with findings of poor performance and management of public hospitals [54, 55], which compose only 4.2% of hospitals. Due to the complexity of healthcare delivery systems with different payment mechanisms, the ALOS and total cost for patients undergoing hip fracture surgery (the performance of hospitals) are influenced by various factors. Because there may be residual confounding due to unobserved factors such as the presence of caregivers and the health status of patients beyond what we measured, further detailed investigations are warranted.

This study demonstrated the feasibility of health system performance comparisons using condition-specific, detailed micro data in East Asia; we developed comparable, nationwide, patient-level health insurance databases using the same protocols across the systems. We also examined not only patient-level data, as most existing studies have done, but also hospital and regional factors related to resource use across countries; the latter are more policy-sensitive and also are factors in which policy makers can intervene. We provided, in a comparative way across the three countries, detailed descriptions of hip fracture surgery patients and the hospitals where they received surgeries. While adjusting for a wide range of covariates, we also confirmed consistent volume-outcome relationships at the hospitals across all three systems with SHI in Asia, similar to those in other regions.

There are also methodological challenges to be overcome to advance health system performance comparison research in the region. The DPC database did not cover all discharged patients in Japan, and patient data could not be linked across different hospitals. For comparability, this study focused on the care at the index hospital of the surgery, which is critical for the path and outcomes of post-acute care. Time to surgery is an interesting variable; however, it was neither available in the Korea and Taiwan data nor the main interest of this study. Further studies are recommended to examine the full episode of hip fracture care focusing on long-term outcomes (e.g., 90- or 180-day readmission or mortality). We only provided descriptive statistics on the in-hospital death variable as the incidence of the event was very low during the index hospitalizations of the population of interest across the three systems. In addition, the three administrative databases differed in the amount of clinical information available, and few could be utilized in the comparative analysis. Finally, there could be discrepancies in coding practice in the secondary data we analyzed.

## Conclusion

This study suggests health system performance widely differs among health systems with similar structural features, which are often the basis for health system typology and comparison. System performance, defined as the efficiency of inpatient care in this study, is more likely to be dependent on how each system operates; there may be room to achieve high-performing health systems through better provision and delivery of care when drastic changes in existing macro-level system features are hardly possible. Different pulling and pushing effects of individual, hospital, and environmental (policy and social context) determinants suggest a need for further investigations into how these factors interact with each other in unique ways within and also across systems. This study also suggests various recent delivery-based reform initiatives in many countries can improve their health system performance. Learning from and benchmarking such innovations is still valid across health systems with similar and even very different financing and governance models. To do so, governments should invest in building comparable heath big data and methods for health system performance comparison (HSPC) as well as collaborative HSPC research networks beyond existing collaborations, which are currently mainly among high-income western countries.

## Data Availability

The data that support the findings of this study are available from the DPC Study Group in Japan for the Japanese data, the National Health Insurance Data Sharing Service in Korea for the Korean data, and the Data Center of the Ministry of Health and Welfare in Taiwan for the Taiwanese data. But restrictions apply to the availability of these data, which were used under license for the current study, and so are not publicly available. Data are, however, available from the authors upon reasonable request and with the permissions of the DPC Study Group in Japan, the National Health Insurance Data Sharing Service in Korea, and the Data Center of the Ministry of Health and Welfare in Taiwan.

## Abbreviations

ALOS: Average length of stay
CCI: Charlson comorbidity index
DPC: Diagnosis Procedure Combination
DRG: Diagnosis-related group
FFS: Fee-for-service
HCQI: Health care quality indicators
HHI: Herfindahl-Hirschman index
HSPC: Health system performance comparison
LOS: Length of stay
NHIS: National Health Insurance Service
SHI: Social health insurance
